# Cutaneous candidiasis mimicking acute generalized exanthematous pustulosis: A case report

**DOI:** 10.1177/2050313X231160949

**Published:** 2023-03-28

**Authors:** Emily Seale, Alison Spurr, Iris Teo, Steven J Glassman

**Affiliations:** 1 Faculty of Medicine, University of Ottawa, Ottawa, ON, Canada; 2 Division of Dermatology, Faculty of Medicine, The Ottawa Hospital, University of Ottawa, Ottawa, ON, Canada; 3 Department of Pathology and Laboratory Medicine, Eastern Ontario Regional Laboratory Association, University of Ottawa, Ottawa, ON, Canada

**Keywords:** Candidiasis, pustulosis, drug eruption, immunosuppression

## Abstract

Disseminated cutaneous candidiasis is a rare manifestation of candidiasis that arises most commonly from *Candida albicans*. It is described as a widespread erythematous papulopustular skin infection that typically affects premature newborns or immunocompromised patients. While candidal infections usually respond well to antifungal therapy, the clinical presentation of disseminated cutaneous candidiasis can often mimic a variety of other dermatologic conditions, that can lead to delayed diagnosis and treatment. We present a 67-year-old comorbid male patient with widespread erythema and superficial pustules resembling acute generalized exanthematous pustulosis (AGEP) that was actually an unexpected manifestation of disseminated cutaneous candidiasis. Prompt initiation of a topical and oral antifungal regimen contributed to marked improvement. Given the high frequency of drug eruptions in comorbid patients receiving multiple medications, alternate diagnoses like infections should be included in the differential diagnosis.

## Introduction

Candidal fungi are considered a normal feature of human microbiota, with approximately 50% of individuals living with candida in their mucosal epithelium.^1^ They are most notably found along the gastrointestinal and genital tracts.^1^ While there are hundreds of species of Candida, a much smaller fraction act as endogenous opportunistic pathogens that adapt their virulence to different environments, especially among immunocompromised patients.^1^ The severity of candidal infections can range from mild mucosal or skin involvement to life-threatening deep organ or hematological disease.^2^ The predominant pathogenic species is *Candida albicans*, and it is found in over 80% of oral fungal cultures around the world.^1^ Disseminated cutaneous candidiasis, also known as candidal pustulosis, is a rare skin manifestation that arises from Candida spp.^3,4^ It is described as a widespread erythematous papulopustular skin infection that arises most often among premature newborns, immunocompromised patients or those with hematologic malignancies.^3,4^ The differential diagnosis of widespread erythema with superficial sterile pustules includes generalized pustular psoriasis (GPP), which is a rare and potentially life-threatening form of psoriasis, and acute generalized exanthematous pustulosis (AGEP), which is typically caused by drug sensitisation.^5^ Herein, we present a 67-year-old comorbid patient with apparent AGEP that was actually a presentation of disseminated cutaneous candidiasis and responded well to a topical and oral antifungals.

## Case

A 67-year-old male with a history of chronic lymphocytic leukemia (CLL), heart failure, type 2 diabetes mellitus, end-stage renal disease on hemodialysis, atrial fibrillation, pulmonary hypertension, and significant peripheral vascular disease with bilateral above-knee amputations presented to the emergency department with a 3-day history of a progressive pruritic pustular eruption and low-grade fever at 38.2°C. He had an elevated white blood cell count of 25.2 x 10^9^/L (normal range: 3.5–10.5 x 10^9^/L) with a neutrophilia of 19.6 x 10^9^/L (normal range: 2.0–7.5 x 10^9^/L), though was known to have a chronic leukocytosis from CLL. He did not have a history of other dermatologic conditions but 3 days prior he had been discharged from a 7-day admission due to a bacteremia related to a scrotal ulcer and pyelonephritis. At that time, he had initially been treated with piperacillin/tazobactam and then stepped down to oral amoxicillin/clavulanate 500 mg daily for 14 days at discharge.

**Figure 1.**
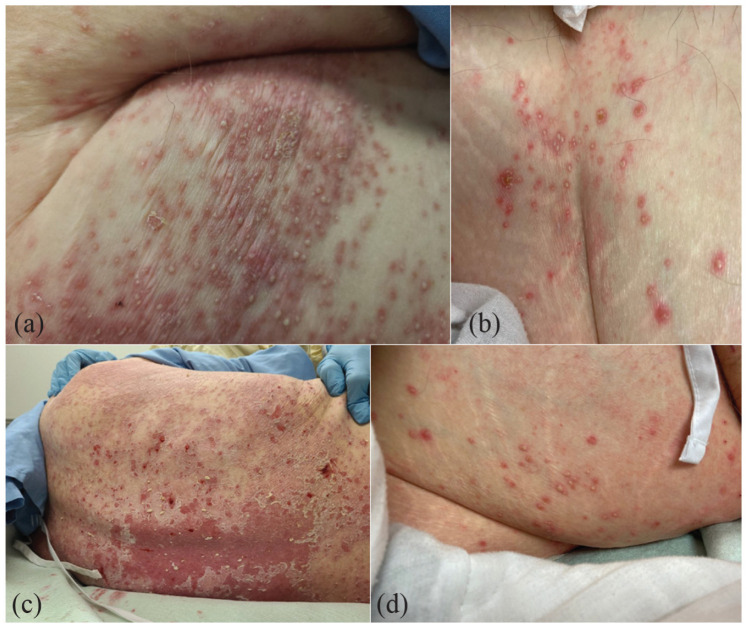
Extensive superficial pustules on a background of erythema, erosion and scale on the (a) Axillary vault, (b) Superior aspect of buttocks, (c) Back and (d) Lateral aspect of buttocks.

On assessment, he had a skin phototype I, with widespread symmetric erythema in folds extending to the back, with innumerable superficial pustules, erosion, and scale (Figure 1(a)–(d)). In addition, there was a large sacral pressure ulcer spreading to the scrotum with surrounding dusky erythema, scale, and pustules.

**Figure 2.**
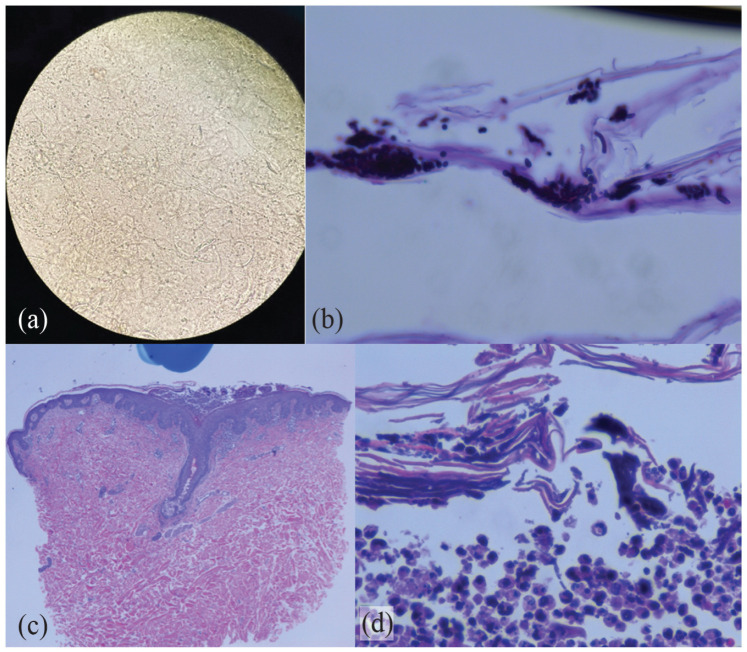
(a) Direct bedside KOH exam revealing branched hyphae. (b) Fungal spores and hyphae on PAS stain (original magnification 600x). (c) Corneal pustule centered over a follicle, with mild superficial perivascular inflammation and acanthosis (H&E, original magnification 40x). (d) Fungal hyphae can be seen within the pustule (H&E, original magnification 400x).

While the initial differential included AGEP from one of his recent antibiotics, a bedside KOH exam of a skin scraping from some pustules revealed branched hyphae, confirming superficial fungal infection, either dermatophyte or Candida spp (Figure 2(a)). This was also noted later in the laboratory by direct microscopy (Figure 2(b)). A punch biopsy of a pustule on the abdomen was also performed for H&E and direct immunofluorescence (DIF) (Figure 2(c) and (d)). It revealed subcorneal and intraepidermal pustules with fungal spores and hyphae, suggestive of a superficial cutaneous fungal infection. DIF was negative. Fungal culture from pustules isolated *Candida albicans/dubliniensis*. Blood culture for Candida spp. was negative. These findings altered the preferred diagnosis to disseminated cutaneous candidiasis, and he was started on oral fluconazole 200 mg daily for 2 weeks, together with clotrimazole cream twice daily. Over the course of 12 days his skin slowly improved.

Given that he was febrile at presentation, blood cultures were taken and were positive for ampicillin-resistant *Klebsiella pneumoniae*, likely secondary to his unresolved scrotal ulcer. Given the concern for AGEP secondary to amoxicillin/clavulanate, it was discontinued, and he was started on IV meropenem 500 mg daily for 3 weeks.

## Discussion

Disseminated cutaneous candidiasis typically presents with diffuse erythematous maculopapular and pustular lesions, which can be accompanied by pruritus or burning. Lesions often start in flexures and then become disseminated.^3,4^ It preferentially affects immunocompromised patients with complex multicomorbidity and polypharmacy use, which can confound the diagnosis.^4^ A broad differential of erythema and superficial pustules should be considered and excluded, including pyogenic infections, GPP, AGEP, miliaria, subcorneal pustular dermatosis, and IgA pemphigus. On history, our patient had recently started a course of amoxicillin/clavulanate, which raised the suspicion for AGEP. Every year, an estimated one to five patients per million develop AGEP, and over 90% of cases are attributed to medications, most commonly antibiotics (including penicillins, cephalosporins, and quinolones).^6^ Interestingly, an increasing incidence of AGEP has been reported since the start of the COVID-19 pandemic, suspected to be a result of the pathogen itself or the new combination of medications introduced for treatment.^7^

In the case of our patient, he also presented with significant multicomorbidities, pressure ulcer and CLL which predisposed him to opportunistic infections including Candida spp. Skin scraping for direct microscopy offered a quick and easy method to confirm superficial fungal infection in this case, permitting rapid diagnosis. Our patient’s fungal culture from the pustules further isolated *C. albicans/dubliniensis. C. albicans* and *C. dubliniensis* are recognized as two highly related opportunistic pathogenic candidal species, with very similar genomes.^8^ Until 1995, C. dubliniensis was referred to as “atypical C. albicans,” but more recently has garnered its own name as differences in virulence-related gene families have been identified. These genetic differences are thought to contribute to *C. albicans’* increased prevalence and pathogenicity among humans, and increased production of hyphae on histopathology, while *C. dubliniensis* tends to be less virulent and develops more adaptive resistance to azole agents.^8^ However, as definite differentiation between *C. albicans* and *C. dubliniensis* remains lengthy, costly, and does not typically influence initial management, these two species are frequently reported together on cultures.^9^ Ongoing research currently seeks to improve the effectiveness and affordability of Candida spp. species differentiation.

With the rise in complex comorbidity, polypharmacy use, and combination antibiotic therapy, it is becoming increasingly challenging to identify and treat the underlying culprit for nonspecific erythematous and pustular cutaneous eruptions. Our case highlights the value of considering a broad differential and combining clinical features, bedside exams and histopathological findings to guide prompt diagnosis and effective management.
